# Geographically distributed data management to support large-scale data analysis

**DOI:** 10.1038/s41598-023-44789-x

**Published:** 2023-10-18

**Authors:** Tamer Z. Emara, Thanh Trinh, Joshua Zhexue Huang

**Affiliations:** 1 Faculty of Computers and Artificial Intelligence, Damietta University, New Damietta, 34519 Egypt; 2 Faculty of Computer Science, Phenikaa University, Ha Dong, 12116 Hanoi, Vietnam; 3 Phenikaa Research and Technology Institute (PRATI), A &A Green Phoenix Group JSC, Cau Giay, 11313 Hanoi, Vietnam; 4 National Engineering Laboratory for Big Data System Computing Technology, Shenzhen University, Shenzhen, 518060 China; 5 Big Data Institute, College of Computer Science and Software Engineering, Shenzhen University, Shenzhen, 518060 China

**Keywords:** Computational platforms and environments, Data mining, Data processing

## Abstract

Nowadays, several companies prefer storing their data on multiple data centers with replication for many reasons. The data that spans various data centers ensures the fastest possible response time for customers and workforces who are geographically separated. It also provides protecting the information from the loss in case a single data center experiences a disaster. However, the amount of data is increasing at a rapid pace, which leads to challenges in storage, analysis, and various processing tasks. In this paper, we propose and design a geographically distributed data management framework to manage the massive data stored and distributed among geo-distributed data centers. The goal of the proposed framework is to enable efficient use of the distributed data blocks for various data analysis tasks. The architecture of the proposed framework is composed of a grid of geo-distributed data centers connected to a data controller (DCtrl). The DCtrl is responsible for organizing and managing the block replicas across the geo-distributed data centers. We use the BDMS system as the installed system on the distributed data centers. BDMS stores the big data file as a set of random sample data blocks, each being a random sample of the whole data file. Then, DCtrl distributes these data blocks into multiple data centers with replication. In analyzing a big data file distributed based on the proposed framework, we randomly select a sample of data blocks replicated from other data centers on any data center. We use simulation results to demonstrate the performance of the proposed framework in big data analysis across geo-distributed data centers.

## Introduction

Cloud computing is getting high attention in the industry and research. Many business organizations and companies have decided to store and process their data in cloud data centers as their collected data is getting bigger and bigger. As the data volume grows exponentially, storing such data within a single data center is no longer achievable. Hence, the need for geographically distributed data centers has been increased. However, the processing for such large amounts of data is quite challenging and would require sufficient storage and computing power^1–5^. More importantly, the development of an intelligent processing method capable of recognizing the meaningful information to understand this data is required.

Computing clusters are the most commonly used to store and process big data. Hadoop^6^ and Spark^7^ are the most popular computing cluster frameworks that emerged for big data processing and analysis^8,9^. However, these frameworks process the big data locally within the same data center^10,11^.

Recently, big data processing over geographically distributed data attracts much attention, especially with the rapid increase of data volumes. It can provide several benefits. For example, Facebook reports in the first quarter of 2020 that the active monthly users are 2.6 billion and over 1.7 billion people use Facebook daily, sharing over 100 billion messages on a daily basis^12^. Also, the number of Stories shared on the platform is over 1 billion. In 2018, Uber^13^ had over 100 petabytes^14^ data that needs to be stored, cleaned, and analyzed with minimum latency, in order to make data-driven decisions at every level, from forecasting rider demand during high traffic events to identifying and addressing the bottlenecks in driver-partner sign-up process.

The traditional method to process large data volumes that are geographically distributed is to copy all the data to a single data center. Then, it can be processed in a centralized fashion^15,16^. However, transferring the complete data to a single data center may not be practically feasible due to the bandwidth limitation, communication and time cost, and data privacy.

The data replication technique plays an important role in distributed systems to increase data safety and availability. The general idea of data replication is to store several copies of a data file on different computing nodes or servers^17^. Also, replicating data into various locations provides fault tolerance. In case a failure happened, another copy can be used to retrieve the lost data^18^. Since availability is improved, the waiting times are reduced, and consequently, the latency is also reduced. A lot of efforts has been spent on utilizing data replication in different systems and technologies such as database systems^19^, parallel and distributed systems^20–23^, data grid systems^24–29^, and mobile systems^30,31^.

Nowadays, several companies prefer storing their data on multiple data centers with replication for many reasons. (1) The data that spans various data centers ensure the fastest possible response time for customers and workforces who are geographically separated^32^. (2) It also protects the information from the loss in case a single data center experiences a disaster^33^. However, the amount of data is increasing at a rapid pace, which leads to challenges in storage, analysis, and various processing tasks.

Recently, we have proposed a high-level architecture design of a big data management system (BDMS)^34^. BDMS stores the big data file as a set of random sample data blocks, each being a random sample of the whole data file^35,36^. BDMS was designed to manage big data files on data block-level in a single computing cluster.

Also, we have recently proposed two data distribution strategies to enable big data analysis across geo-distributed data centers^37^. Both strategies target at the companies that consider two scenarios to store their big data in multiple data centers either without replication or with replications. In this paper, we focus on the second strategy that endeavors to store data with replication. We propose a data management framework to manage the distributed data among geo-distributed data centers. The proposed architecture is composed of a grid of geo-distributed data centers (GDCs) connected to a data controller (DCtrl). The DCtrl is responsible for organizing and managing the block replicas across the geo-distributed data centers. We use the BDMS system as the installed system on the distributed data centers. The goal of the proposed framework is to enable efficient use of the distributed data blocks for various data analysis tasks.

To demonstrate the performance of big data analysis based on the proposed framework, we built a simulation environment to simulate a grid of five data centers. We used this environment to conduct several experiments. The experimental results show that a small set of block samples is enough to get an approximate result of the whole data.

The remainder of this paper is organized as follows. “Related work” section discusses the related works. The proposed geo-distributed data management framework is introduced in “Geo-distributed data management framework (GDDM)” section. “Replication management” section discusses how the proposed framework manages the block replicas. “Geo-distributed ensemble learning application” section introduces the geo-distributed ensemble learning application to analyze the managed data by the proposed framework. In “Simulation results” section, we illustrate the results of our experiments. Finally, “Conclusions” section concludes the paper.

## Related works

Data replications technique is widely used in current distributed file systems to protect the stored data against failure or data loss, such as Hadoop Distributed File System (HDFS)^38^, Google File System (GFS)^39,40^ and others. In HDFS, the default replication factor is 3. When a client requests to write a file, the first data block is written in the same DataNode used by the client. The other two replicas are stored in different DataNodes in different racks.

Due to the rapid increase of cloud computing storage systems, Data replication techniques attract much attention to achieve high availability and reliability. In Ref.^41^, an algorithm was proposed to optimize the replication cost using the concept of the knapsack problem. The main idea of this algorithm is to estimate the replication cost. When the cost exceeds the user budget, the replicas are transferred to a lower-cost data center. Liu and Shen^42^ proposed a popularity-aware multi-failure resilient and cost-effective replication (PMCR) scheme. It divides the cloud storage system into two tiers, the primary tier and the backup tier. To manage the correlated and independent failures, the data is replicated on two servers in the primary tier and one server in the backup tier. Its simulation results showed that PMCR guarantees high data availability and durability.

Data locality is a key factor improving the performance of geo-distributed big data application. The performance of accessing remote data is slower than accessing local data. The remote data access may be acceptable for rarely-accessed data. In contrast, it slows down the performance for frequently-used data. To cope with such issue, dynamic data replication^43^ was proposed. Dynamic data replication strategies mainly rely on creating replicas of frequently accessed data close to the user devices.

Many efforts have been made to propose and develop different dynamic data replication algorithms, such as^17,44–46^. A prefetching-aware data replication (PDR) strategy was proposed in Ref.^17^ to prefetch the most popular files based on the correlations of the data files in the file access history. It consists of three stages. It first builds a dependency matrix through calculating the dependencies between all files. Then, it determines the most popular file according to the total average of file accesses. Finally, it replaces the unnecessary replicas with the more popular replicas to save the storage space of each node. To leverage the decentralized architecture of decentralized storage systems such as Dynamo^47^ or Voldemort^48^, Matri et al.^44^ proposed a write-enabled dynamic replication scheme.

In summary, the above works focus on facilitating the creation of data replicas to save the cost and reduce the execution time. Also, they consider the replication on the file level. In this paper, we propose and design a geo-distributed data management framework. The proposed framework manages the big data files on the block-level. Therefore, it is doing replication on the block-level instead of the file-level. Besides, it supports block-level sampling to approximate the data analysis of the distributed data across geo-distributed data centers.

## Geo-distributed data management framework (GDDM)

**Figure 1:**
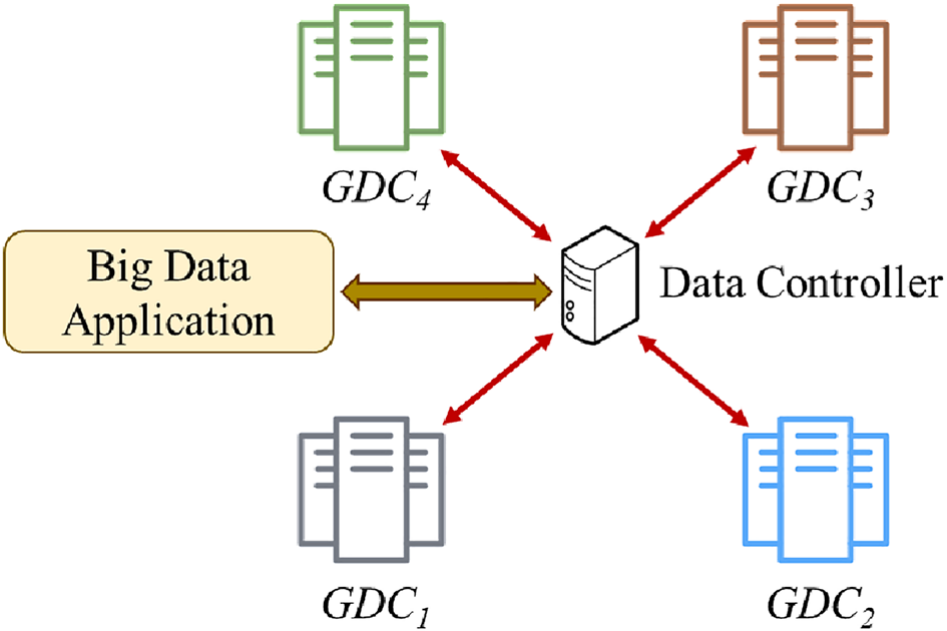
Geo-distributed data management framework.

In this section, we discuss the proposed architecture of the geo-distributed data management (GDDM) framework. The main objective of the GDDM framework is to enable and manage data replication across geographically distributed data centers. The proposed architecture is composed of a grid of geo-distributed data centers (GDCs) connected to a data controller (DCtrl) as shown in Fig. 1.

### Data controller (DCtrl)

The DCtrl is responsible for managing the block replicas across the geo-distributed data centers. The main component of DCtrl is the *replica manager* which is composed of three parts: replica placement, replica replacement, and replica selection. Replica placement builds and stores the replication table; it determines the best possible location to store data files based on user request and network protocol. Replica replacement determines which replica to be replaced for a new one when the storage is full or restricted. Replica selection picks the proper GDC location that has the required data file for the big data application.

DCtrl organizes the data blocks on the geo-distributed data centers using a hierarchy namespace of files and directories. The namespace tree starts with an identifier of GDC (GDC-ID) followed by the namespace that is issued by the BDMS system for the data file. The client does not need to know how or where data is stored or manipulated. In fact, the cloud systems provide the environment that the user does not need to know precisely the location of a specific file or service and their delivery process while hosting their application at the time that the cloud service provider controls the entire service.

DCtrl stores the namespace of each data file and the list of blocks belonging to it which comprises the metadata of the name system in the *replica-image*. Besides, DCtrl stores any modifications to the replica-image in a log file. When a DCtrl restarts, it restores the namespace by reading the replica-image and replaying the log file.

### Geo-distributed data centers (GDCs)

GDC refers to the geo-distributed data center. We use the BDMS system^34^ to store the data files on different data centers. BDMS stores data files as random sample data blocks. Suppose a big data application (BDA) needs to write a big data file. It first requests the location of the closest GDC from the DCtrl. The BDA then writes the data into the closest GDC which in turn stores the written data as random sample data blocks. After that, the closest GDC pushes the data in a pipeline which is organized from the replicas’ locations ordered by their proximity to the first replica.

Furthermore, GDC has an important component named *voting table*. Clients vote for files at sites close to them. For instance, an application requests reading a file if this file is not in the closest GDC, the application votes for this file at the closest GDC. After that, DCtrl uses this table in replicating the files dynamically. Also, GDC builds another component named *frequent table*. Frequent table stores the Block-ID and frequent number. The frequent number refers to the number of times that the replica is requested by a BDA. Replica replacement uses the frequent table to move the least frequent number replica to another location in case the storage becomes full.

When a GDC starts, it connects first to the DCtrl and performs a *handshake*. The handshake aims to verify the GDC-ID and the metadata of the current GDC. If the DCtrl knows the GDC-ID, it joins the grid system. Otherwise, it will not be able to join the system. When a GDC is newly formatted and initialized, it needs to register with the DCtrl to associate an ID. Also, the DCtrl classifies the newly connected GDC to a particular grid cluster.

The GDC sends periodically a *block report* to the DCtrl. The block report contains information about the blocks, that the GDC hosts, such as *block-id*, *time generation stamp*, and the length of the block replica. After GDC registration, it sends immediately the first block report. The successive block reports are sent periodically to provide the DCtrl with an up-to-date view of the location of the block replicas.

During the normal operation, the GDCs send *heartbeats* to DCtrl in order to confirm that the GDC is being live and operating well as well as the hosted block replicas by the GDC are available.

### Grid clustering

**Figure 2:**
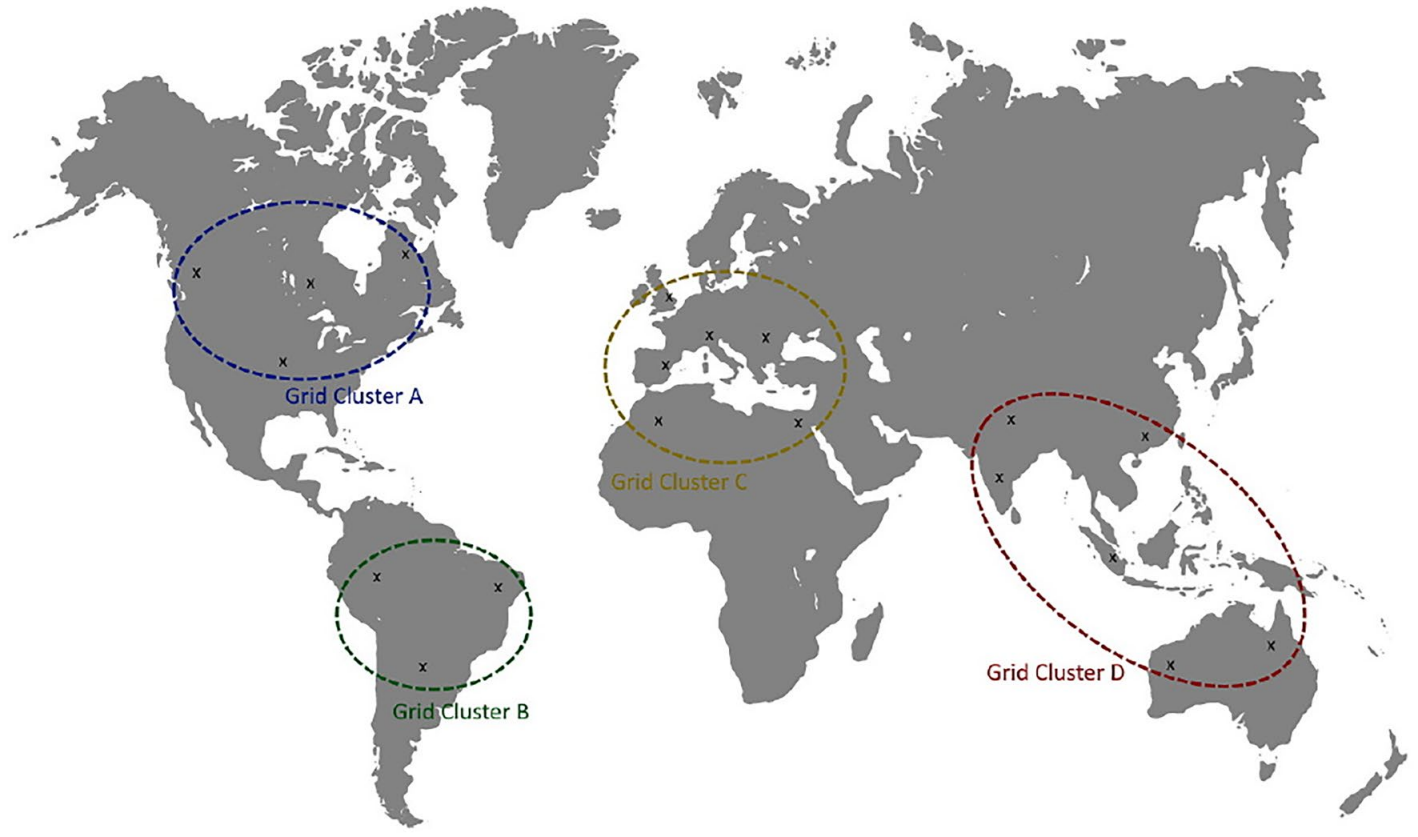
An example to demonstrate the grid clustering process.

The first step of DCtrl work is to cluster the geographically distributed data centers into $\gamma$ clusters, where $\gamma$ is the replication factor. The produced clusters are used in replica placement where each replica is stored in a separate grid cluster. The objective of this operation is to ensure that the replicas are distributed in balanced distances and the user can find the needed data in a near location. Figure 2 shows an example demonstrating the grid clustering process using $\gamma = 4$.

K-means is used in the clustering of GDCs and the separation of the retained clusters based on the length of their centroids using the Haversine formula (Eq. 1) of the great-circle distance between two points^49^:

1 $$\begin{aligned} D=2R \arcsin {\left( \sqrt{ sin^2 \left( \frac{\phi _1 - \phi _2}{2} \right) + \cos {\phi _1} \cos \phi _2 \sin ^2 \left( \frac{\lambda _1 - \lambda _2}{2} \right) }\right) }, \end{aligned}$$

where *D* is the distance (in km) between two points on the earth identified by latitude $\phi$ and longitude $\lambda$ (in radians) and *R* is the radius of the earth (in km); here, the geometric mean was used, that is, 6367.45 km.

### Balancer

The replica placement strategy, used by the replica manager in GDDM, does not take into account the disk space utilization of GDCs. Data might not be placed uniformly across different GDCs which causes imbalance. Furthermore, imbalance occurs when new GDCs are connected to the grid.

The balancer works in a similar way as the HDFS’s balancer tool. It balances disk space usage on every grid cluster. It considers a threshold value as an input parameter. The threshold value is a fraction between (0, 1). The balancer moves replicas between the GDCs in the same grid cluster until it is deemed to be balanced, which means that the utilization of every GDC differs from the utilization of the grid cluster by no more than the given threshold parameter.

This tool iteratively moves replicas from GDCs with lower free disk space to other GDCs with higher free disk space in the same grid cluster. In order to maintain data availability, the balancer uses the replica replacement strategy discussed in “Replica replacement” section where the replicas with the least frequent number are chosen to move. The destination is selected based on the voting number where the location of the highest voting number is the recommended location. Moreover, it is worth mentioning that the balancer does not reduce or increase the number of replicas while it just changes the location of the replicas.

### Replica scanner

Replica scanner is a tool on every GDC. It periodically scans all replicas and verifies that the stored checksum equivalents the replica data. Besides, when a user reads a block replica, it first verifies its checksum. In case that the verification succeeds, it informs the GDC. In the same time, GDC considers it as a verification of the replica and no need to run the replica scanner tool on this block replica.

Whenever a replica scanner or a read user detects a corrupt block replica, it notifies the DCtrl. The DCtrl marks the block replica as a corrupt replica, but it does not remove the block replica immediately. Instead, it initiates to copy a good replica of the block. When the count of the good replicas reaches the replication factor, the corrupt replica can be removed. The goal of this restriction is to preserve data as long as possible. Suppose that all replicas of a block are corrupt; this restriction allows the user to retrieve the data from the corrupt replicas.

## Replication management

The main objective of DCtrl is to ensure that each data block has several replicas distributed into different data centers. The DCtrl receives a report from the different GDCs when a block replica arrives. Using this report, the DCtrl detects the block that has become over- or under-replicated. When a block becomes over-replicated, the DCtrl reduces the number of the replicas by selecting a replica to remove. Also, when a block becomes under-replicated due to a failure happened or data loss, the DCtrl selects the best possible location to store the replica. The goal is to balance the distance between the different block replicas in order to increase data availability. The DCtrl manages the replicas through three parts:

### Replica placement

When a DCtrl starts, it launches the grid clustering as discussed in “Grid clustering” section to produce separate GDCs clusters. Replica placement uses these GDCs clusters to transfer the new block replicas where each block replica is transferred to a separate GDCs cluster. This step guarantees that the distances between the different replicas of the same block are approximately equal. Therefore, wherever a client is, he can find a close replica increasing data availability.

**Figure 3:**
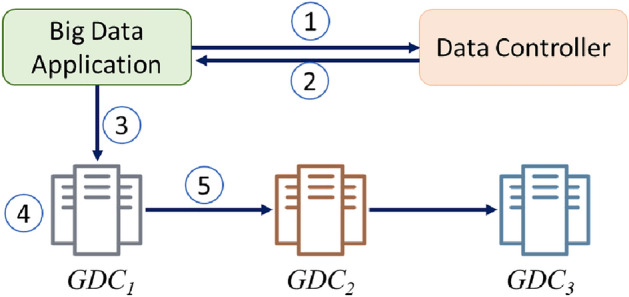
The writing process flow.

When a client requests writing a file, the DCtrl places the first replica at the closest GDC to the client. In this way, the DCtrl aims to minimize the write cost and increase data availability. The other replicas are distributed to the other GDCs clusters. DCtrl selects the best possible location inside every GDCs cluster to transfer the replica. After all target locations are selected, the selected GDCs are organized as a pipeline ordered by their closeness to the first replica. Figure 3 shows the details of the writing data process. The numbered arrows describe the execution flow triggered by a top-level read job initiated by a big data application. The main steps to write a big data file are summarized as follows:

1. BDA initializes a connection with the DCtrl and requests DCtrl to nominate a set of $\gamma$ locations to host the replicas, where $\gamma$ is the replication factor.
2. The DCtrl replies with a set of $\gamma$ locations. The first location is the closest GDC to BDA location at all and then other locations are in the order of their closeness to the first replica.
3. BDA transfers the data to the closest GDC.
4. The BDMS of the closest GDC converts and stores the data as random sample data blocks, each being a random sample of the whole data.
5. The closest GDC organizes a pipeline of other GDCs in the order of their closeness. Then, the data are pushed in this order to the other data centers.

**Figure 4:**
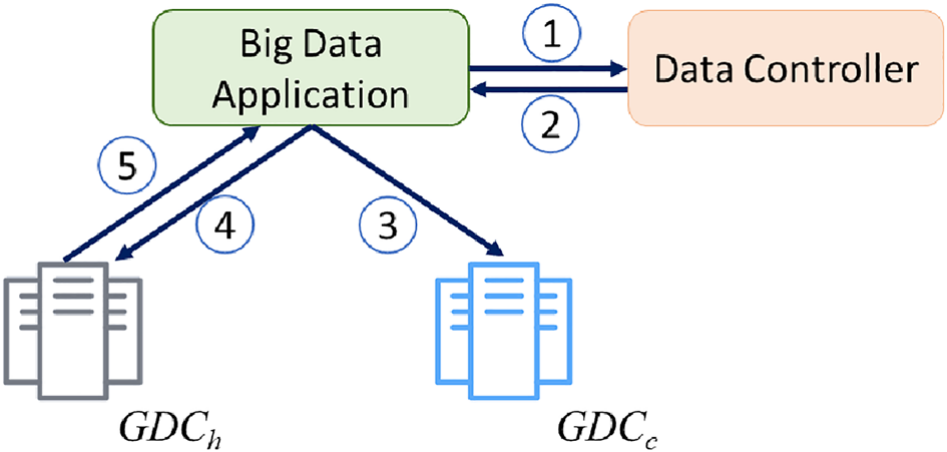
The reading process flow.

For the reading process, the DCtrl sends the block locations to the client ordered by their closeness to the reader. Figure 4 shows the details of a block replica reading process. The numbered arrows describe the execution flow triggered by a top-level read job. A big data application (BDA) wanting to read a file first contacts the DCtrl to get the best location of data blocks making up the file and then reads block contents from the closest GDC. The main steps of reading a file by a big data application are summarized as follows:

1. BDA initializes a connection with the DCtrl and requests the closest GDC storing the required data.
2. DCtrl replies with the information of the closet GDC hosting the block replica ($GDC_h$) and the closest GDC at all ($GDC_c$).
3. If $GDC_h$ is not $GDC_c$, the BDA votes at $GDC_c$ for the required block replica.
4. BDA requests the required data from $GDC_h$.
5. $GDC_h$ replies with the required block replica. Also, $GDC_h$ increments the frequent number of the requested block replica in the frequent table.

### Replica replacement

The DCtrl receives periodically a block report from the different GDCs. If the DCtrl detects that a block replica becomes over-replicated, it selects a replica to remove. The DCtrl removes the replica with the least frequent number. When a block replica becomes under-replicated, the missing replicas are copied to the locations of the highest voting number of the same block.

In case that the storage is full, the DCtrl moves the block replica with the least frequent number to another location. The other location is determined based on the voting number of the same block in the different GDCS. The DCtrl selects the location of the highest voting number to increase the availability and reduce the bandwidth utilization.

### Replica selection

When a big data application requests a data file to perform a job execution, the DCtrl chooses the appropriate replica location to execute the job. The DCtrl estimates the bandwidth between the two places. It selects the appropriate location based on several parameters such as bandwidth, network protocol, memory usage, and distance. Besides, BDMS provides the statistical summary of the blocks, such as the number of records, the number of features, mean, variance, max, min, and among others. For specific analysis tasks, DCtrl can select particular blocks based on these specific statistical features.

## Geo-distributed ensemble learning application

In machine learning, ensemble learning refers to learning methods that use multiple models built with one or multiple learning algorithms from multiple component data sets in an ensemble model to gain better performance in classification or prediction than any single model built with one algorithm from one training data set. Random samples are widely used in ensemble learning to obtain multiple component data sets from a given training data set.

**Figure 5:**
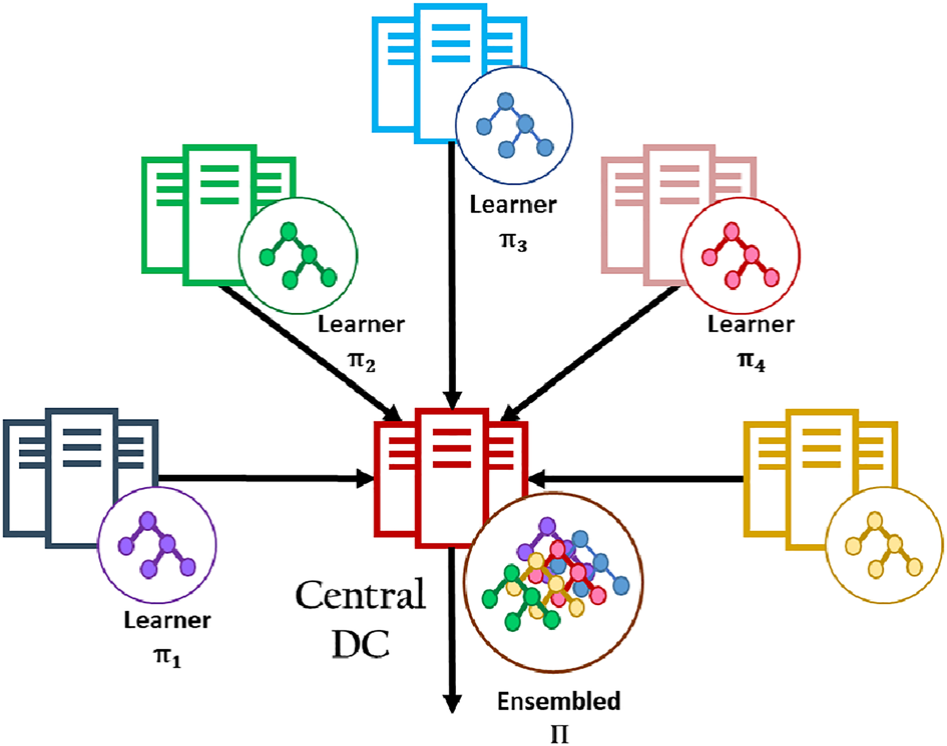
The geo-distributed ensemble learning application: for each data center, a learning algorithm is applied on a subset of RSP blocks which is selected randomly to build a base learner. After that, the learner models are sent to the central data center to build the ensemble model.

The geo-distributed data analysis framework is illustrated in Fig. 5. Suppose that a big dataset *D* is distributed across 5 data centers. Using GDDM, The first operation is to partition the data stored on each data center into a set of random sample data blocks. Next step is the data replication. When each of them replicates its data on other data centers, the end result, the stored data on each data center can be considered as a random sample of *D*. Next, for each data center, a base learner is created by training a model on a randomly selected subset of the data from each data center. For instance, four learners $\pi _1, \pi _2, \pi _3, \pi _4$ are built in parallel, as shown in Fig. 5. Finally, each data center sends the learner model to the central data center to build the ensemble model $\Pi$.

The geo-distributed data analysis framework is applicable to many learning tasks, including estimation of statistics of a big data set *D*, supervised and unsupervised learning. In future, we will investigate different learning tasks using the geo-distributed data analysis framework.

## Simulation results

In this section, we use simulation results to show the performance of a geo-distributed ensemble model in building classification models. These experiments were conducted on a cluster consisting of 5 nodes. Each node has 12 cores (24 with Hyper-threading), 128 GB RAM, and 12.5 TB disk storage. The operating system is Ubuntu version 14.04.5. Apache Hadoop 2.6.0 and Apache Spark 2.3.0 are installed on this cluster. Scala version 2.11.12 is used for the implementation.

### Data generation

In the experiments, we generated two big data sets with the form $$ Z_i=(X_{i}, Y_{k})$$, with the identical and independent distribution for $$i = \{1,..., N\}$$, $$\forall Y_k \in (Y_1,...,Y_K): X_{i,m}=N(\mu _{k,m},\sigma _{k,m})$$, where $$m = \{1, ..., M\}$$, $$\mu _{k,m}\in U\left( 0,10\right) $$ and $$\sigma _{k,m} \in U\left( 0,10\right) $$. We generated both data sets with the following parameters: the number of features (*M*) = 100, the number of classes (*K*) = 500, and the number of records (*N*) = 100, 000, 000. The final generated data volume was 100 GB, and the data records are sorted in class labels. The difference between both datasets is the distribution of the class labels. DS1 has 500 classes distributed equally; i.e. all classes have an equal number of objects. On the other hand, the probability distribution of the class labels of DS2 is unbalanced with classes (from class label 1 to class label 100) make $$ 70\% $$ of the total number of the objects.

We assume that the data are distributed into 5 data centers. Therefore, we divided each data set (DS1, and DS2) into 5 data subsets, {D1, D2, D3, D4, D5}, equally and randomly and then converted to RSP data blocks using RRPlib^50^.

### Performance of the geo-distributed ensemble learning model

In this section, we demonstrate the performances of data analysis across geo-distributed data centers using the geo-distributed ensemble model. We use three learning algorithms, decision tree, random forest, and logistic regression, in building the classification models. We recorded the performances of execution time and classification accuracy of models built from random samples of the distributed data centers. In order to simulate the performance of the geo-distributed ensemble learning model on multiple data centers, we use the following steps to summarize the experiment procedures for DS1 and DS2:

1. Suppose that there are five data centers, each having the same configurations as our university’s computing cluster. Each data center stores a data subset of the generated data {D1, D2, D3, D4, D5}.
2. In order to simulate the data replication technique, we build new five subsets {DC1, DC2, DC3, DC4, DC5}. Each block in old subsets is replicated to a random three new subsets as the replication factor is 3. The new five data sets simulated the data stored in each data center after data replication.
3. We built a classification model for each produced data subset from some samples of the data. We used decision tree algorithm to build a model from DC1. Random forest algorithm is used to build models from DC2 and DC3. Logistic regression algorithm is used to build models from DC4 and DC5.
4. After building the five models, we used the voting method as a consensus function to predict the final class label.
5. We repeated the experiment 20 times and then reported the average of the accuracy and the processing time.

**Figure 6:**
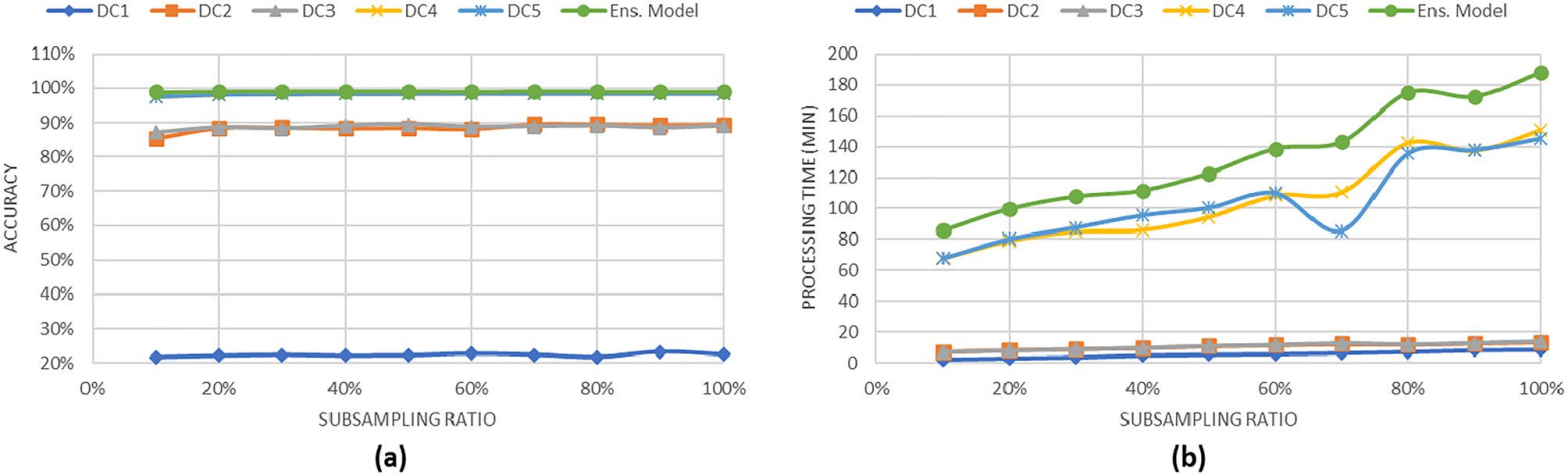
Classification results for the synthesized data set DS1 which is distributed into 5 subsets.

**Figure 7:**
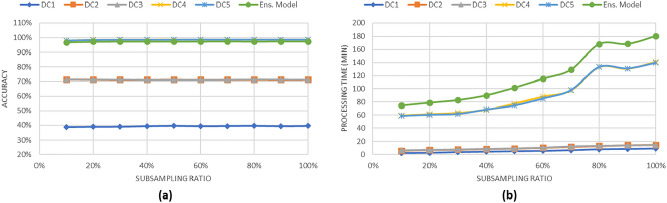
Classification results for the synthesized data set DS2 which is distributed into 5 subsets.

Figures 6 and 7 show the results of the classification tasks using geo-distributed ensemble model. As shown in both figures, when 20% of the data is used to build the ensemble models, the accuracy is approximately 99% in DS1 case and 97% in DS2 case. Moreover, the processing time for classifying 20% of the data in both figures is less than 100 min without including the transferring time between data centers. The increase of the processing time is due to the processing time used by logistic regression algorithm. In this experiment, we assume the data is already replicated to the various data centers at generation time; therefore, we neglect the time consumed on replication.

## Conclusions

In this paper, we have proposed a data management framework to mange the distributed data among geo-distributed data centers. We have discussed the design and architecture of the proposed framework. The proposed architecture is composed of a grid of geo-distributed data centers connected to a data controller. The data controller manages and organizes the blocks replicas across the geo-distributed data centers. The proposed framework supports large-scale ensemble model data analysis. The experimental results show that a sample of the data on each data center is enough to be a representative of the whole distributed data.

## Data Availability

The datasets used and/or analysed during the current study available from the corresponding author on reasonable request.

## Abbreviations

BDA: Big data application
BDMS: Big data management system
DCtrl: Data controller
GDC: Geo-distributed data center
GDC-ID: Identifier of geo-distributed data center
GDCs: Geo-Distributed Data Centers
GDDM: Geo-distributed data management framework
HDFS: Hadoop distributed file system
